# Genes, Cells and Brain Areas of Intelligence

**DOI:** 10.3389/fnhum.2019.00044

**Published:** 2019-02-15

**Authors:** Natalia A. Goriounova, Huibert D. Mansvelder

**Affiliations:** Department of Integrative Neurophysiology, Center for Neurogenomics and Cognitive Research, Neuroscience Amsterdam, VU University Amsterdam, Amsterdam, Netherlands

**Keywords:** intelligence, temporal cortex, frontal cortex, pyramidal cells, dendrites, GWAS of gene expression, action potentials

## Abstract

What is the neurobiological basis of human intelligence? The brains of some people seem to be more efficient than those of others. Understanding the biological foundations of these differences is of great interest to basic and applied neuroscience. Somehow, the secret must lie in the cells in our brain with which we think. However, at present, research into the neurobiology of intelligence is divided between two main strategies: brain imaging studies investigate macroscopic brain structure and function to identify brain areas involved in intelligence, while genetic associations studies aim to pinpoint genes and genetic loci associated with intelligence. Nothing is known about how properties of brain cells relate to intelligence. The emergence of transcriptomics and cellular neuroscience of intelligence might, however, provide a third strategy and bridge the gap between identified genes for intelligence and brain function and structure. Here, we discuss the latest developments in the search for the biological basis of intelligence. In particular, the recent availability of very large cohorts with hundreds of thousands of individuals have propelled exciting developments in the genetics of intelligence. Furthermore, we discuss the first studies that show that specific populations of brain cells associate with intelligence. Finally, we highlight how specific genes that have been identified generate cellular properties associated with intelligence and may ultimately explain structure and function of the brain areas involved. Thereby, the road is paved for a cellular understanding of intelligence, which will provide a conceptual scaffold for understanding how the constellation of identified genes benefit cellular functions that support intelligence.

## What Is Intelligence?

Intuitively we all know what it is to be intelligent, although definitions of intelligence can be very diverse. It is something that helps us plan, reason, solve problems, quickly learn, think on our feet, make decisions and, ultimately, survive in the fast, modern world. To capture this elusive trait, cognitive tests have been designed to measure performance in different cognitive domains, such as processing speed and language. Very soon it became clear that the results of different cognitive tests are highly correlated and generate a strong general factor that underlies different capabilities—general intelligence or Spearman’s *g* (Spearman, 1904). One of the most used tests nowadays to estimate Spearman’s *g* is the Wechsler Adult Intelligent Scale (WAIS). This test combines results of multiple cognitive tests in one measurement, full-scale IQ score.

Are the tests able to measure human intelligence and does expressing it in a single number—IQ score—make sense? Despite critiques of this reductionist approach to intelligence, the tests have proven their validity and relevance. First, results of IQ tests strongly correlate with life outcomes, including socioeconomic status and cognitive ability, even when measured early on in life (Foverskov et al., 2017). The increasing complexity and technology-dependent society imposes ever growing cognitive demands on individuals in almost every aspect of everyday life, such as banking, using maps and transportation schedules, reading and understanding forms, interpreting news articles. Higher intelligence offers many seemingly small advantages, but they accumulate to affect overall chances in life of individuals (Gottfredson, 1997). These are beneficial to socioeconomic status, education, social mobility, job performance, and even lifestyle choices and longevity (Lam et al., 2017).

Second, intelligence turns out to be a very stable trait from young to old age in the same individual. In a large longitudinal study of English children, a correlation of 0.81 was observed between intelligence at 11 years of age and scores on national tests of educational achievement 5 years later. This contribution of intelligence was evident in all 25 academic disciplines (Deary et al., 2007). Even at much later age, intelligence remains stable: a single test of general intelligence taken at age 11 correlated highly with the results of the test at the age of 90 (Deary et al., 2013).

Finally, one of the most remarkable findings of twin studies is that heritability of intelligence is extraordinarily large, in the range 50%–80% even reaching 86% for verbal IQ (Posthuma et al., 2001). This makes human intelligence one of the most heritable behavioral traits (Plomin and Deary, 2015). Moreover, with every generation, assortative mating infuses additive genetic variance into the population, contributing to this high heritability (Plomin and Deary, 2015).

Thus, despite its elusiveness in definition, intelligence lies at the core of individual differences among humans. It can be measured by cognitive tests and the results of such tests have proven their validity and relevance: intelligence measures are stable overtime, show high heritability and predict major life outcomes.

## Biological Basis of Intelligence: A Whole-Brain Perspective

### Are Bigger Brains Smarter?

A question that has puzzled scientists for centuries is that of the origin of human intelligence. What makes some people smarter than others? The quest to answer these questions has started as early as 1830s in Europe and Russia where the brains of deceased elite scientists and artists were systematically collected and meticulously studied (Vein and Maat-Schieman, 2008). However, all the attempts to dissect the exceptional ability and talent did not reveal much at that time.

The reigning hypothesis of the past century was that smarter people have bigger brains. With the advances in neuroimaging techniques this hypothesis was put to test in many studies. Indeed, a meta-analysis of 37 studies with over 1,500 individuals of the relationship between *in vivo* brain volume and intelligence found a moderate, yet significant positive correlation of 0.33 (McDaniel, 2005). A more recent meta-study of 88 studies with over 8,000 individuals again reported a significant, positive, slightly smaller correlation coefficient of 0.24. One of the conclusions of this study was that the strength of the association of brain volume and IQ seems to be overestimated in the literature but remains robust after accounting for publication bias (Pietschnig et al., 2015). Thus, overall bigger brain volume, when analyzed across multiple studies, is associated with higher intelligence.

### Which Brain Areas Are Important for Intelligence?

**Figure 1:**
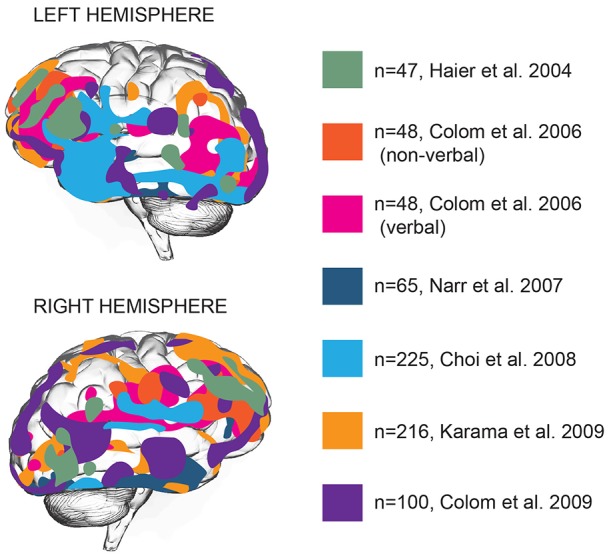
Gray matter thickness of multiple cortical areas correlates with general intelligence. Brain areas with significant association between cortical thickness and general intelligence in different studies are represented by different colors. *N* numbers represent sample sizes. In all cases the areas correlating with general intelligence are shown, with the exception of the Colom et al.’s (2006) study, where verbal and non-verbal intelligence were reported separately (Haier et al., 2004; Colom et al., 2006, 2009; Narr et al., 2007; Choi et al., 2008; Karama et al., 2009).

Brain function is distributed across various areas that harbor specific functions. Can intelligence be attributed to one or several of these areas? Structural and functional brain imaging studies focused on locating general intelligence within the brain and linking specific types of cognition to specific brain areas (Deary et al., 2010). Early imaging studies associating intelligence to brain structure showed that full-scale IQ scores, a measure of general intelligence, showed a widely distributed pattern of correlations with brain structures: IQ scores correlated with intracranial, cerebral, temporal lobe, hippocampal, and cerebellar volumes (Andreasen et al., 1993), that together encompass almost all brain areas. Voxel-based morphometry (VBM), a neuroimaging analysis technique that allows estimation of focal differences in brain structure, makes it possible to test whether any such areas are clustered together or distributed throughout the brain. Application of VBM to brain imaging data revealed that positive correlations between intelligence and cortical thickness are located primarily in multiple association areas of frontal and temporal lobes (Hulshoff Pol et al., 2006; Narr et al., 2007; Choi et al., 2008; Karama et al., 2009). Based on 37 neuroimaging studies, Jung and Haier (2007) put forward that in particular the structure of frontal Brodmann areas 10, 45–47, parietal areas 39 and 40, and temporal area 21 positively contribute to IQ scores (Jung and Haier, 2007). This model was extended by later studies to frontal eye field, orbitofrontal area, as well as a large number of areas in temporal lobe—inferior and middle temporal gyrus, parahippocampal cortex and auditory association cortex (Narr et al., 2007; Choi et al., 2008; Colom et al., 2009; Figure 1).

### Brain Structure Changes

Brain structure is not fixed at one particular developmental time point and then remains unaltered for the rest of our lives. Gray matter volume changes throughout childhood as well as adulthood (Gogtay et al., 2004) and is influenced by learning, hormonal differences, experience and age. Gray matter changes may reflect rearrangements of dendrites and synapses between neurons (Gogtay et al., 2004). When people acquire a new skill, for instance juggling, transient and selective structural changes are observed in brain areas that are associated with the processing and storage of complex visual motion (Draganski et al., 2004). Similarly, sex differences and age differences are important factors that influence brain structure and can affect which cortical areas associate with intelligence.

Substantial sex differences were reported in the pattern of correlations between intelligence and regional gray and white matter volumes (Haier et al., 2005; Narr et al., 2007; Yang et al., 2014; Ryman et al., 2016), but the reports do not fully agree on the brain areas showing sex differences or their association with cognitive performance. Haier et al. (2005) reported correlations of IQ with parietal and frontal regions in males, whereas women showed correlations mainly within the frontal lobe (Haier et al., 2005). Similar results were obtained by Ryman et al. (2016) in males—fronto-parietal gray matter was more significantly related to general cognitive ability. However, in females the results indicated associations with intelligence in white matter efficiency and total gray matter volume (Ryman et al., 2016). Yet different conclusions were drawn by Narr et al. (2007), where women showed significant associations in gray matter thickness in prefrontal and temporal association cortices, whereas men show associations primarily in temporal-occipital association cortices (Narr et al., 2007). Finally, in a recent study where surface-based morphometry (SBM) was applied instead of VBM, substantial group differences in brain structure were found between sexes but cognitive performance was unrelated to brain structural variation within and between sexes (Escorial et al., 2015).

What the studies do agree on is that substantial sex differences exist in brain structure, but that these differences not always underlie variation in cognitive performance. For example, one of the well-established sex differences in brain structure is the increased cortical thickness of males compared to females (Lüders et al., 2002), but relationships between full-scale IQ score and brain tissue volumes do not differ between men and women (Narr et al., 2007; Escorial et al., 2015).

### Age Matters

In addition to sex differences, gray matter volume shows dramatic changes during lifetime that are part of normal development (Gogtay et al., 2004). The initial increase at earlier ages is followed by sustained thinning around puberty. This developmental change is thought to be a result of overproduction of synapses in early childhood and increased synaptic pruning in adolescence and young adulthood (Bourgeois et al., 1994). Furthermore, different areas have their own timeline of maturation: higher-order association cortices mature only after lower-order somatosensory and visual cortices (Gogtay et al., 2004). Correlations with intelligence follow a similar developmental curve. The strongest correlations between gray matter volume and intelligence have been found for children around the age of 10 years (Shaw et al., 2006; Jung and Haier, 2007). However, at age 12, around the start of cortical thinning, a negative relationship emerges (Brouwer et al., 2014). Moreover, it seems that the whole pattern of cortical maturation unfolds differently in more intelligent children. Children with higher IQ demonstrate a particularly plastic cortex, with an initial accelerated and prolonged phase of cortical increase and equally vigorous cortical thinning by early adolescence (Shaw et al., 2006).

### Brain Specialization to Different Types of Intelligence

In addition to associations of cortical structure with intelligence, imaging studies have revealed correlations of functional activation of cortical areas with intelligence. Psychology distinguishes between two types of intelligence that together comprise Spearman’s *g*: crystallized and fluid intelligence. Crystallized intelligence is based on prior knowledge and experience and reflects verbal cognition, while fluid intelligence requires adaptive reasoning in novel situations (Carroll, 1993; Engle et al., 1999).

Multiple studies imply that fluid intelligence relies on more efficient function of distributed cortical areas (Duncan et al., 2000; Jung and Haier, 2007; Choi et al., 2008). In particular, lateral frontal cortex, with its well-established role in reasoning, attention and working memory, seems to support fluid intelligence, but also the parietal lobe is implicated. One of the earlier studies of fluid intelligence using Raven’s Advanced Progressive Matrices by Haier et al. (1988) demonstrated activation of several areas in the left-hemisphere, in particular posterior cortex. Cognitive performance showed significant negative correlations with cortical metabolic rates, suggesting more efficient neural circuits (Haier et al., 1988). In later studies, fluid intelligence was strongly linked to both function and structure of frontal lobe regions (Choi et al., 2008). When participants perform verbal and nonverbal versions of a challenging working-memory task, while their brain activity is measured using functional magnetic resonance imaging (fMRI), individuals with higher fluid intelligence are more accurate and have greater event-related neural activity in lateral prefrontal and parietal regions (Gray et al., 2003). Also in a PET-scan study, participants showed a selective recruitment of lateral frontal cortex during more complicated cognitive tasks compared to easier tasks (Duncan et al., 2000). In a more recent report, the measurements of gray matter volume of two frontal areas—orbito-frontal (OFC) and rostral anterior cingulate cortices (rACC)—were complemented by white matter connectivity between these regions. Together, left gray matter volume and white matter connectivity between left posterior OFC and rACC accounted for up to 50% of the variance in general intelligence. Thus, especially in prefrontal cortex, structure, function and connectivity all relate to general intelligence, specifically to reasoning ability and working memory (Ohtani et al., 2014).

Crystallized intelligence that largely relies on verbal ability, on the other hand, depends more on the cortical structure and cortical thickness in lateral areas of temporal lobes and temporal pole (Choi et al., 2008; Colom et al., 2009). While parietal areas (Brodman area 40) show overlap in their involvement in crystallized and other types of intelligence, temporal Brodman area 38 is exclusively involved in crystallized intelligence. These findings harmonize well with the function of the temporal lobe—it is thought to be responsible for integrating diverse semantic information from distinct brain regions. Studies of patients with semantic dementia support the role of temporal lobe in semantic working memory as well as memory storage (Gainotti, 2006).

Thus, subdividing Spearman’s *g* reveals distinct cortical distributions involved in subdomains of intelligence. It is likely that further subdividing fluid and crystallized intelligence, for instance in verbal comprehension, working memory, processing speed, and perceptual organization, may result in a more defined map of cortical regions on left and right hemisphere that relate to these subdomains of intelligence (Jung and Haier, 2007).

### White Matter and Intelligence

Not only gray matter, but also white matter volumes show an association with intelligence that can be explained by common genetic origin (Posthuma et al., 2002). White matter consists of myelinated axons transferring information from one brain region to another and integrity of the white matter tracts is essential for normal cognitive function. Thus, specific patterns of white matter dysconnectivity are associated with heritable general cognitive and psychopathology factors (Alnæs et al., 2018). For example, Yu et al. (2008) found that mental retardation patients show extensive damage in the integrity of white matter tracts that was assessed by fractional anisotropy. IQ scores significantly correlated with the integrity of multiple white matter tracts in both healthy controls and mental retardation patients (Yu et al., 2008). This correlation was especially prominent in right uncinate fasciculus that connects parts of temporal lobe with the frontal lobe areas (Yu et al., 2008). These results support previous findings on the association of particularly temporal and frontal lobe gray matter volume and intelligence (Hulshoff Pol et al., 2006; Narr et al., 2007; Choi et al., 2008; Karama et al., 2009) and emphasize that intact connectivity between these areas is important for intelligence.

Longitudinal studies that track changes in white matter across development and during aging also show that changes in white matter are accompanied by changes in intelligence. During brain maturation in children, white matter structure shows associations with intelligence. In a large sample (*n* = 778) of 6- to 10-year-old children, white matter microstructure was linked to non-verbal intelligence and to visuospatial ability, independent of age (Muetzel et al., 2015). In another study, where white matter was studied in typically-developing children vs. struggling learners, the white matter connectome efficiency was strongly associated with intelligence and educational attainment in both groups (Bathelt et al., 2018).

Also at later stages in life, changes in white matter microstructure are coupled with changes in intelligence (Ritchie et al., 2015). Substantial correlations of 12 major white matter tracts with general intelligence were found in older individuals (Penke et al., 2012). Subsequent analysis showed that lower white matter tract integrity exerts a substantial negative effect on general intelligence through reduced information-processing speed (Penke et al., 2012). Thus, structurally intact axonal fibers across the brain provide the neuroanatomical infrastructure for fast information processing within widespread brain networks, supporting general intelligence (Penke et al., 2012).

### Conclusions on Gross Brain Distribution of Intelligence

Thus, both functional and structural neuroimaging studies show that general intelligence cannot be attributed to one specific region. Rather, intelligence is supported by a distributed network of brain regions in many, if not all, higher-order association cortices, also known as parietal-frontal network (Jung and Haier, 2007; Figure 1). This network includes a large number of regions—the dorsolateral prefrontal cortex, the parietal lobe, and the anterior cingulate, multiple regions within the temporal and occipital lobes and, finally, major white matter tracts. Some limited division of function can be observed, implicating frontal and parietal areas in fluid intelligence, temporal lobes in crystallized intelligence and white matter integrity in processing speed.

Although brain imaging studies have identified anatomical and functional correlates of human intelligence, the actual correlation coefficients have consistently been modest, around 0.15–0.35 (Hulshoff Pol et al., 2006; Narr et al., 2007; Choi et al., 2008; Karama et al., 2009). There are most likely various reasons for this, but an important conclusion is that human intelligence can only partly be explained by brain structure and functional activation of cortical areas observed in MRI. There are other factors contributing to intelligence that have to be considered. To put it in an evolutionary perspective, the human brain has outstanding cognitive capabilities compared to other species, that include many specific human abilities—abstract thinking, language and creativity. However, human brain anatomy is not that distinct from other mammalian species and it cannot satisfactorily account for a marked evolutionary jump in intelligence. Both in its size and neuronal count, the human brain does not evolutionary stand out: elephants and whales have larger brains (Manger et al., 2013) and long-finned pilot whale cortex contains more neurons (37 billion) than that of humans (19–23 billion; Pakkenberg and Gundersen, 1997; Herculano-Houzel, 2012; Mortensen et al., 2014). Especially the brains of our closest neighbors on the evolutionary scale, non-human primates, show remarkable resemblance. In fact, the human brain is anatomically in every way a linearly scaled-up primate brain (Herculano-Houzel, 2012), and appears to have little exceptional or extraordinary features to which outstanding cognitive abilities can be attributed. Thus, answers to the origins of human intelligence and its variation between individuals most probably do not lie only in the gross anatomy of the brain, but rather should be sought at the level of its building blocks and computational units—neurons, synapses and their genetic make-up.

## A Genetic Approach to Intelligence

Given that intelligence is one of the most heritable traits, it follows that also its neurobiological correlates should be under strong genetic influence. Indeed, both cortical gray and white matter show a gradient of similarity in subjects with increasing genetic affinity (Thompson et al., 2001; Posthuma et al., 2002). This structural brain similarity is especially strong in frontal and lateral temporal regions, which show most significant heritability (Thompson et al., 2001). Hence, overall brain volume links to intelligence and to a large extent shares a common genetic origin. How and when during the development is genetic influence exerted by individual genes and what are the genes that determine human intelligence?

### Genes of Intelligence

Over the last decade, genome-wide association studies (GWAS) evolved into a powerful tool for investigating the genes underlying variation in many human traits and diseases (Bush and Moore, 2012). GWAS studies test for associations between phenotypes and genetic variants—single-nucleotide polymorphisms (SNPs)—in large groups of unrelated individuals. Although the large majority of SNPs have a minimal impact on biological pathways, some SNPs can also have functional consequences, causing amino acid changes and thus lead to the identification of genetic underpinnings of a disease or a trait (Bush and Moore, 2012).

After the first wave of GWAS of intelligence studies yielded mostly non-replicable results (Butcher et al., 2008; Davies et al., 2011, 2015, 2016; Trampush et al., 2017) it became evident that intelligence is a highly polygenic trait and much larger sample sizes are needed to reliably identify contributing genes (Plomin and von Stumm, 2018). Meta-analysis of the first 31 cohorts (*N* = 53,949) could only predict ~1.2% of the variance in general cognitive function in an independent sample and biological pathway analysis did not produce significant findings (Davies et al., 2015). Using educational attainment as proxy phenotype of intelligence boosted both the sample size and the number of found associated genes. Educational attainment is the number of years spent in full-time education. Both phenotypically (Deary et al., 2010) and genetically (Trampush et al., 2017) it strongly correlates with IQ. Because the number of school years is one of the common, routinely gathered parameters, this approach increased sample sizes to ~400,000 individuals in the latest GWAS studies (Okbay et al., 2016). Even larger samples sizes were obtained by combining the GWAS for cognitive ability with educational attainment (Lam et al., 2017; Trampush et al., 2017) and by focusing on GWAS of intelligence in multiple cohorts (Savage et al., 2018; Zabaneh et al., 2018). What are the genes of intelligence identified by these studies?

### Intelligence Is a Polygenic Trait

The latest and largest genetic association study of intelligence to date identified 206 genomic loci and implicated 1,041 genes, adding 191 novel loci and 963 novel genes to previously associated with cognitive ability (Savage et al., 2018). These findings show that intelligence is a highly polygenic trait where many different genes would exert extremely small, if any, influence, most probably at different stages of development. Indeed, the reported effect sizes for each allele are extremely small (generally less than 0.1% for even the strongest effects), and the combined effects genome-wide explain only a small proportion of the total variance (Lam et al., 2017). For example, the strongest effect of identified alleles on educational attainment explains only 0.022% of phenotypic variance in the replication sample (Okbay et al., 2016), and the combined effects genome-wide predict only a small proportion of the total variance in hold-out samples (Lam et al., 2017). At the same time, the overall SNP heritability reported in recent GWAS is around 20%–21%, (Lam et al., 2017; Trampush et al., 2017; Savage et al., 2018; Coleman et al., 2019), less than half of the heritability estimates in twin studies (>50%; Plomin and von Stumm, 2018). However, small genetic effects at critical stages of development may have large consequences on brain function and development and together with it on cognitive ability. Thus, it is important to know what these identified genes are, but also when and where they are expressed in the nervous tissue.

### Most SNPs Found in Non-coding Regions

Non-coding regions comprise most of the human genome and harbor a significant fraction of risk alleles for neuropsychiatric disease and behavioral traits. Over the last decade, more than 1,200 GWAS studies have identified nearly 6,500 disease- or trait-predisposing SNPs, but only 7% of these are located in protein-coding regions (Pennisi, 2011). The remaining 93% are located within non-coding regions, suggesting that GWAS-associated SNPs regulate gene transcription levels rather than altering the protein-coding sequence or protein structure.

**Figure 2:**
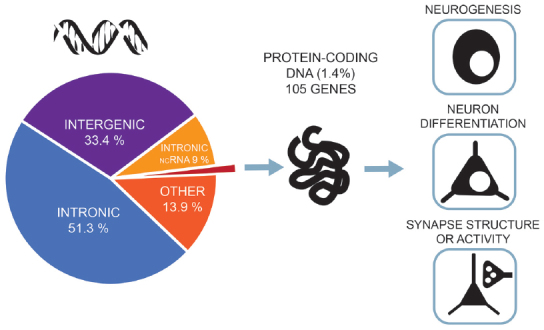
Most of the associated genetic variants of intelligence lie in non-coding DNA regions—only 1.4% of the associated single-nucleotide polymorphisms (SNPs) are exonic, non-synonymous variants and lie in protein-coding genes. Gene-set analyses implicate pathways related to neurogenesis, neuron differentiation and synaptic structure. The figure is based on the results from the most recent and largest genome-wide association studies (GWAS) of intelligence by Savage et al. (2018).

A very similar picture emerges for GWAS of intelligence studies. SNPs significantly associated with intelligence are mostly located in intronic (51.3%) and intergenic areas (33.4%), while only 1.4% are exonic (Savage et al., 2018; Figure 2). Similar distributions were also found in earlier association studies (Sniekers et al., 2017; Coleman et al., 2019). However, it is exactly these non-coding, gene regulatory regions that make the genome responsive to changes in synaptic activity and constitute a major force behind the evolution of human cognitive ability (Hardingham et al., 2018). While the function of most intergenic regions in human DNA remain poorly defined, new insights emerge from studies combining high-resolution mapping of non-coding elements, chromatin accessibility and gene expression profiles. These studies link the regulatory elements to their target genes. Thus, neurogenesis and cortical expansion in humans is thought to be controlled by specific genetic regulatory elements—human-gained enhancers (HGEs), that show increased activity in the human lineage (de la Torre-Ubieta et al., 2018). Moreover, genetic variants associated with educational attainment were shown to be enriched within the regulatory elements involved in cortical neurogenesis (de la Torre-Ubieta et al., 2018).

Thus, genetic effects on cognitive ability most probably do not operate independently of environmental factors, but rather reveal themselves through signal-regulated transcription driven by experience. This interplay between the epigenetic effects through regulatory elements and genetic make-up would also explain the increasing heritability of intelligence with age (Bergen et al., 2007; Davis et al., 2008; Plomin and Deary, 2015). The same regulatory genes require proper gene-environment interactions to reveal their role in cognitive ability. In other words, during development, the same set of genes acquires an increasing impact on intelligence as early levels of cognitive ability become reinforced through the selection of environments and education consistent with those ability levels (Briley and Tucker-Drob, 2013; Plomin and von Stumm, 2018).

### Most Genes Are Active During Neurodevelopment

Many GWAS results identify genes and biological pathways that are primarily active during distinct stages of prenatal brain development (Bergen et al., 2007; Okbay et al., 2016; Lam et al., 2017; Sniekers et al., 2017; Trampush et al., 2017). A number of these genes were previously implicated in intellectual disability or developmental delay (Coleman et al., 2019). Specifically, some genes with known mutations of large effect in mental disease show smaller regulatory effects on cognition, indicating naturally occurring dose-response curves regarding gene function (Trampush et al., 2017; Coleman et al., 2019).

Combining the SNP-data with transcriptome data showed that the candidate genes exhibit above-baseline expression in the brain throughout life, but show particularly higher expression levels in the brain during prenatal development (Okbay et al., 2016). When genes were grouped into functional clusters, many such clusters associated with educational attainment are primarily involved in different stages of neural development: the proliferation of neural progenitor cells and their specialization, the migration of new neurons to the different layers of the cortex, the projection of axons from neurons to their signaling target and dendritic sprouting (Okbay et al., 2016). Also for intelligence, gene-set analysis identifies neurogenesis, neuronal differentiation and regulation of nervous system development as major functions of the identified SNPs (Savage et al., 2018; Figure 2).

Some examples from the latest GWAS of intelligence involve genes with known functions in cell proliferation and mitosis: the GNL3 gene is involved in stem cell proliferation, NCAPG stabilizes chromosomes during mitosis, and DDX27 alters RNA secondary structure and is involved in embryogenesis, cellular growth and division (NCBI Resource Coordinators, 2017; Savage et al., 2018). Finally, the largest and most significantly enriched cluster of genes associated with educational attainment contains genes with transcription cofactor activity (Okbay et al., 2016), supporting the role of candidate genes in neurodevelopment and regulation of gene expression. Indeed, many protein-coding genes, identified in the latest GWAS of intelligence, produce products that contain DNA and RNA interacting domains, such as Zink fingers and RING finger domains (ZNF446, MZF1, ZNFX1, ZNF638, RNF123), or known RNA binding partners (RBFOX and CELF4; NCBI Resource Coordinators, 2017; Savage et al., 2018).

### Genes Involved in Cell-Cell Interactions

Many of the identified genes that play a role in neurodevelopment might contribute to synaptic function and plasticity. Brain function relies on highly dynamic, activity-dependent processes that switch on and off genes. These can lead to profound structural and functional changes and involve formation of new and elimination of unused synapses, changes in cytoskeleton, receptor mobility and energy metabolism. Cognitive ability may depend on how efficient neurons can regulate these processes. Interactions of cells with their direct environment is a fundamental function in both neurodevelopment and synaptic function. Many of the top protein-coding genes associated with cognitive ability are membrane-anchored proteins responsible for cell-to-cell and cell-to-matrix communication. For example, the ITIH3 gene that codes for a protein that stabilizes the extracellular matrix. Another example is LAMB2 gene that codes for laminin, an extracellular matrix glycoprotein a major constituent of basement membranes. Also several cadherin genes, PCDHA1 to PCDHA7, CDHR4, that are involved in cell adhesion, associate with cognitive ability (NCBI Resource Coordinators, 2017; Savage et al., 2018). In addition, in an extremely high IQ cohort, the gene most significantly enriched for association is ADAM12, a membrane-anchored protein involved in cell–cell and cell–matrix interactions (Zabaneh et al., 2018). Finally, some candidate genes that code for cell adhesion molecules (DCC and SEMA3F; Savage et al., 2018) are specifically involved in axon guidance during neuronal development.

Some candidate genes are involved in the regulation of different signaling pathways through surface receptors. Such examples involve DMXL2 that regulates the Notch signaling pathway; SPPL2C signal peptide peptidase like 2C, RNF43 ring finger protein 43 that negatively regulates Wnt signaling pathways (Savage et al., 2018) and the WNT4 gene that encodes secreted signaling proteins (Sniekers et al., 2017; Coleman et al., 2019). These signaling pathways play an essential role in embryogenesis, cell proliferation, migration, but also synaptic communication throughout development.

Remarkably, recent large-scale cellular-resolution gene profiling has identified species-specific differences exactly in the same functional categories of genes involved in intercellular communication (Zeng et al., 2012). By contrasting mouse and human gene expression profiles in neocortex, the cross-species differences in gene expression included secreted protein (48%), extracellular matrix (50%), cell adhesion (36%), and peptide ligand (31%) genes. These results may highlight the importance of cell-to-environment interactions not only for human intelligence but also for human evolution in general.

### Genes of Synaptic Function and Plasticity

Some findings of GWAS of intelligence point directly at genes with known functions in synaptic communication, plasticity and neuronal excitability. Some identified genes are primarily involved in presynaptic organization and vesicle release. One of those is TSNARE1 that codes for t-SNARE domain containing 1 (Savage et al., 2018). The primary role of SNARE proteins is to mediate docking of synaptic vesicles with the presynaptic membrane in neurons and vesicle fusion (NCBI Resource Coordinators, 2017). Furthermore, at least two other identified genes are also involved in vesicle trafficking: GBF1 mediates vesicular trafficking in Golgi apparatus and ARHGAP27 plays a role in clathrin-mediated endocytosis. Finally, BSN gene codes for a scaffolding protein involved in organizing the presynaptic cytoskeleton.

One of the transcriptional activators associated with intelligence is cAMP responsive element binding 3L4 (CREB3L4). This gene encodes a CREB—a nuclear protein that modulates the transcription of genes. It is an important component of intracellular signaling events and has widespread biological functions. However, in neurons its most documented and well-studied roles is the regulation of synaptic plasticity, learning and memory formation (Silva et al., 1998).

Tapping into databases of drug targets and their gene annotations can shed new light on the associations of drug gene-sets with a phenotype (Gaspar and Breen, 2017). Such a drug pathway analysis combined with GWAS results of intelligence revealed that the gene targets of two drugs involved in synaptic regulation and neuron excitability were significantly enriched: a T-type calcium channel blocker and a potassium channel inhibitor (Lam et al., 2017). In a related analysis of drug classes, significant enrichment was also observed for voltage-gated calcium channel subunits (Lam et al., 2017). In another study, genes involved in regulation of voltage-gated calcium channel complex were also significantly linked to educational attainment in a previous study (Okbay et al., 2016). Both ion channel types play a critical role in synaptic communication and action potential firing. T-type calcium channels are involved in action potential initiation and switching between distinct modes of firing (Cain and Snutch, 2010). Potassium channels are crucial for rapid repolarization during AP generation and maintenance of a resting membrane potential (Hodgkin and Huxley, 1952).

### Genes With Supporting Functions

The human brain uses at least 20% of the entire body’s energy consumption. Most of this energy demand goes to generation postsynaptic potentials (Attwell and Laughlin, 2001; Magistretti and Allaman, 2015). Notably, the emergence of higher cognitive functions in humans during evolution is also associated with the increased expression of energy metabolism genes (Magistretti and Allaman, 2015). Genes involved in energy supply and metabolism could thus have an impact on maintenance of high-frequency firing during cognitive tasks. Indeed, cognitive ability associates with genetic variation in several genes that code for regulators of mitochondrial function—GPD2, NDUFS3, MTCH2 (NCBI Resource Coordinators, 2017; Savage et al., 2018).

Mitochondria are central for various cellular processes that include energy metabolism, intracellular calcium signaling, and generation of reactive oxygen species. By adapting their function to the demands of neuronal activity, they play an essential role in complex behavior of neurons (Kann and Kovács, 2007). In addition, genes involved in lipid metabolism (BTN2A1 and BTN1A1) and glucose and amino acid metabolism (GPT) are among the candidate genes of intelligence.

Another remarkable cluster of protein-coding genes implicated in intelligence are genes coding for microtubule-associated proteins. Microtubules are an essential part of the cytoskeleton and are involved in maintaining cell structure throughout development. At the same time, microtubules are important highways of intracellular transport, and thereby affect recycling of synaptic receptors and neurotransmitter release in neurons (Hernández and Ávila, 2017). The MAPT gene coding for microtubule-associated protein was linked to intelligence by several studies (Sniekers et al., 2017; Trampush et al., 2017; Savage et al., 2018; Coleman et al., 2019). MAPT is also altered in many brain diseases—Alzheimer’s disease, Parkinson’s disease and Huntington’s disease (Hernández and Ávila, 2017). Apart from MAPT, some other genes coding for microtubule associated proteins were found to be significantly associated with intelligence: microtubule associated serine/threonine kinase 3 (MAST3), ALMS1 functions in microtubule organization and SAXO2 (FAM154B) a microtubule-stabilizing protein (NCBI Resource Coordinators, 2017; Savage et al., 2018).

### Conclusions From Genetic Studies

In conclusion, twin studies show that individual differences in human intelligence can largely (50%–80%) be explained by genetic influences making intelligence one of the most heritable traits. However, present GWAS studies can capture less than half of this heritability (21%–22%; Lam et al., 2017; Trampush et al., 2017; Savage et al., 2018; Coleman et al., 2019). Furthermore, genetic influences are attributed to miniscule effects by a large number of genes. Ninety-five percent of these genetic variants are located in intronic and intergenic regions and might have a gene regulatory function. Only a very small proportion of associated SNPs (1.4%), are located in DNA fragments that are translated into protein.

The majority of associated genes are implicated in early, most probably prenatal development, with some genes essential for synaptic function and plasticity throughout lifespan. The fact that such traits as birth length/weight and longevity show robust polygenic correlations with cognitive performance (Lam et al., 2017; Trampush et al., 2017) implies that overall healthy development is a prerequisite for optimal cognitive function.

GWAS tests possible associations between genes and phenotype. However, the availability of cell-type and tissue-specific transcriptome data from post-mortem human brains (Ardlie et al., 2015) has opened a new horizon for GWAS studies. Linking hits of GWAS data to cell-type and tissue-specific transcriptomic profiles (GTEx) may indicate in which brain region and even which cell types intelligence genes are potentially expressed. This approach has obvious caveats, since genes associated with intelligence do not have to be expressed at the same developmental time, and since brain loci involved in intelligence are widely distributed, not all genes need to be expressed in the same brain area or cell type. Nevertheless, using this approach, it was found that genes associated with educational attainment and intelligence preferentially express together in nervous tissue (Okbay et al., 2016; Lam et al., 2017; Trampush et al., 2017; Savage et al., 2018; Coleman et al., 2019). Specifically, hippocampal, midbrain and generally cortical and frontal cortical regions show the highest enrichment of expression of these genes (Savage et al., 2018; Coleman et al., 2019). With the exception of midbrain, these are brain regions previously implicated in intelligence by brain imaging studies.

Cell-type specific expression profiles of genes of intelligence highlight the role of neuronal cell types. Although glia cells are the most abundant cell type in the human brain (Vasile et al., 2017), no evidence for enrichment of candidate genes in oligodendrocytes or astrocytes was found (Lam et al., 2017; Trampush et al., 2017) leaving neurons as the main carrier of genetic variation. Further in-depth analysis of neuronal types revealed significant enrichment of associated genes within pyramidal neurons in hippocampal area CA1 and cortical somatosensory regions. In addition, significant associations were found in the principal cell type in striatum—the medium spiny neurons (Savage et al., 2018; Coleman et al., 2019). Pyramidal neurons are the most abundant neuronal types in neocortex and hippocampus, structures associated with higher executive functions, decision-making, problem-solving and memory. Striatal medium spiny neurons constitute 95% of all neuronal types within the striatum, a structure responsible for motivation, reward, habit learning and behavioral output (Volkow et al., 2017). The results of the GWAS studies put forward the hypothesis that these neuron types play a role in supporting intelligence (Coleman et al., 2019). Is there evidence that particular properties of brain cells contribute to intelligence?

## Cells of Intelligence

Ever since Ramón y Cajal postulated his neuron doctrine of information processing calling neurons “butterflies of the soul” (Cajal, 1893), neuroscience has agreed that the basis of human intelligence must lie in neurons or networks of neurons. However, the neuroscientific search for the biological basis of intelligence has so far focused almost exclusively on the macroscopic brain level and genetics of intelligence, leaving a large gap of knowledge at cellular level.

We assume that our mind functions through the activity of 86 billion neurons (Herculano-Houzel, 2012) and their connections, that form principal building blocks for coding, processing, and storage of information in the brain and ultimately give rise to cognition (Salinas and Sejnowski, 2001). Given the astronomic number of neuronal connections (Drachman, 2005), even the slightest change in efficiency of information processing by neurons can translate into large differences in cognitive ability. Indeed, one of the most robust and replicable associations in behavioral psychology is that of intelligence with mental processing speed, measured by reaction times by human test subjects (Vernon, 1983; Barrett et al., 1986). However, very few studies attempted to answer the question whether the activity and structure of single human neurons support human intelligence and how faster mental processing can be brought about by properties of cells in our brain.

This knowledge gap is not surprising: the access to neurons in the living human brain is very limited and most of what is known about the function of neurons comes from laboratory animal research. During the past decades, the use of brain tissue resected during neurosurgical treatment of epilepsy or tumors has opened new avenues for studying the human brain on the cellular level (Molnár et al., 2008; Testa-Silva et al., 2010, 2014; Verhoog et al., 2013, 2016). To gain access to affected deep brain structures, neurosurgeons resect overlaying non-pathological neocortex that can be transported to the lab for further investigation. In combination with cognitive testing prior to surgery, this approach offers great opportunity to study neuronal function in relation to human intelligence. Such use of living human brain tissue from neurosurgery cannot be substituted by other techniques: post-mortem tissue is generally not suitable for physiological studies (but see Kramvis et al., 2018), while brain imaging studies lack the necessary cellular precision.

### The Key Role of Pyramidal Neurons

Genetic studies indicate that expression of genes associated with intelligence accumulates in cortical pyramidal neurons (Savage et al., 2018; Coleman et al., 2019). Comparisons of key cellular properties of pyramidal neurons across species may offer insights into functional significance of such differences for human cognition. In fact, human tissue used in research always comes from higher-order association areas, typically temporal cortex, in order to spare primary sensory and language functions of the patient. These are exactly the areas implicated by brain imaging in human intelligence. Which properties of pyramidal neurons from temporal cortex stand out when compared across species?

First, the structure of pyramidal cells is different (Elston and Fujita, 2014): compared to rodents and macaques, human layer 2/3 pyramidal cells have threefold larger and more complex dendrites (Mohan et al., 2015). Moreover, these large dendrites also receive two times more synapses than rodent pyramidal neurons (DeFelipe et al., 2002).

Apart from structural differences, human pyramidal neurons display a number of unique functional properties. human excitatory synapses recover 3–4 times faster from depression than synapses in rodent cortex, have more speedy action potentials and transfer information at up to nine times higher rate than mouse synapse (Testa-Silva et al., 2014). In addition, adult human neurons can associate synaptic events in a much wider temporal window for plasticity (Testa-Silva et al., 2010; Verhoog et al., 2013). These differences across species may suggest evolutionary pressure on both dendritic structure and neuronal function in temporal lobe and emphasize specific adaptations of human pyramidal cells in cognitive functions these brain areas perform.

**Figure 3:**
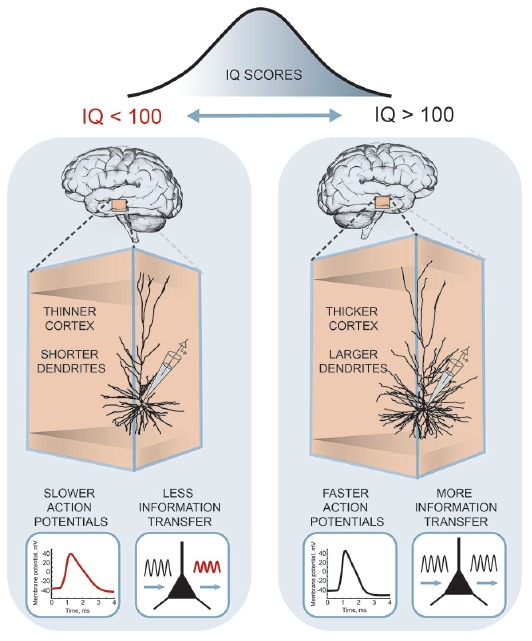
A cellular basis of human intelligence. Higher IQ scores associate with larger dendrites, faster action potentials during neuronal activity and more efficient information tracking in pyramidal neurons of temporal cortex. The figure is based on the results from Goriounova et al. (2018).

Recently, these differences in human pyramidal neuron function and structure were linked to the intelligence scores and anatomical structure of temporal lobes from the same subjects (Goriounova et al., 2018; Figure 3). The results showed that high IQ scores associated with larger temporal cortical thickness in neurosurgery patients, as in healthy subjects (Choi et al., 2008). Furthermore, thicker temporal cortex linked to larger, more complex dendrites of human pyramidal neurons. Incorporating these realistic dendritic morphologies into computational model showed that larger model neurons were able to process synaptic inputs with higher temporal precision. Improved information transfer by model neurons was due to faster action potentials in larger cells. Finally, as predicted by the model, experimental recordings of action potential spiking in human pyramidal neurons demonstrated that individuals with higher IQ scores were able to sustain fast action potentials during neuronal activity. These findings provide the first evidence that human intelligence is associated with larger and more complex neurons and faster action potentials and more efficient synaptic information transfer (Goriounova et al., 2018).

### Connecting Levels: Genes, Cells, Networks and Brain Areas

Pyramidal cells, especially in superficial layers of multimodal integration areas such as temporal or frontal cortex, are main integrators and accumulators of synaptic information. Larger dendrites can physically contain more synaptic contacts and process more information. Indeed, dendrites of human pyramidal neuron receive twice as many synapses than those in rodents (DeFelipe et al., 2002). The increasing information integration capacity of these brain areas is also reflected in a gradient in complexity of pyramidal cells across cortical areas—cells have increasingly larger dendrites in regions involved in higher-order cortical processing (Elston et al., 2001; Jacobs et al., 2001; Elston, 2003; Elston and Fujita, 2014; van den Heuvel et al., 2015). Both in humans and other primates, cortico-cortical whole-brain connectivity positively correlates with the size of pyramidal cell dendrites (Scholtens et al., 2014; van den Heuvel et al., 2015).

Overall, larger dendritic length in human neurons compared to other species, and in particular elongation of their basal dendritic terminals (Deitcher et al., 2017) would enable these cells to use branches of their dendritic tree as independent computational compartments. Recently, Eyal et al. (2016, 2018) have provided new insights into signal processing and computational capabilities of the human pyramidal cells by testing their detailed models including excitatory synapses, dendritic spines, dendritic NMDA- and somatic spikes (Eyal et al., 2018). The results show that particularly large number of basal dendrites in human pyramidal cells and elongation of their terminals compared to other species result in electrical decoupling of the basal terminals from each other. Similar observations were also recently made by dendritic recordings from human layer 5 pyramidal neurons (Beaulieu-Laroche et al., 2018). In this way, human dendrites can function as multiple, semi-independent subunits and generate more dendritic NMDA- spikes independently and simultaneously, compared to rat temporal cortex (Eyal et al., 2014). Dendritic spikes through NMDA receptors are an essential component of behaviorally relevant computations in neurons. In mice, manipulation of these spikes lead to decreased orientation selectivity of visual cortical neurons linking the function of dendrites to visual information processing by neurons (Smith et al., 2013). Furthermore, larger dendrites have an impact on excitability of cells (Vetter et al., 2001; Bekkers and Häusser, 2007) and determine the shape and rapidity of action potentials (Eyal et al., 2014). Increasing the size of dendritic compartments *in silico* lead to acceleration of action potential onset and increased encoding capability of neurons (Eyal et al., 2014; Goriounova et al., 2018). In addition, compared to mouse, human pyramidal neurons in superficial layers show more hyperpolarization activated currents that facilitate excitability of these cells (Kalmbach et al., 2018).

Thus, larger dendrites equip cells with many computational advantages necessary for rapid and efficient integration of large amounts of information. The fact that the larger and faster human neurons in temporal cortex link to intelligence (Goriounova et al., 2018) provides evidence that there is a continuum of these cellular properties across the human population. At the high end of the IQ score distribution, pyramidal cells of individuals with high IQ receive more synaptic inputs and are able to achieve higher resolution of synaptic integration by processing these multiple synaptic inputs separately and simultaneously. As cells are constantly bombarded by a large load of incoming signals during cognitive activity, the neuron has to relay these multiple inputs into output. Human neurons of individuals with higher IQ are able to translate these inputs into action potentials—output signal of the cell—much more efficiently, transfer more information and sustain fast action potential firing compared to lower IQ subjects. These findings harmonize well with genetic and imaging studies identifying metabolic rate as an important correlate of intelligence (Haier et al., 1988; Savage et al., 2018).

Finally, genetic studies of intelligence also implicate genes supporting dendritic structure in human cognitive ability. Clustering of candidate genes from GWAS of educational attainment in gene sets with known biological function identified gene sets involved in cerebral cortex morphology and specifically in dendrites and dendritic spine organization (Okbay et al., 2016). Furthermore, the strongest emerging genetic association with intelligence established by Sniekers et al. (2017) and later replicated in a much larger sample (Coleman et al., 2019) is in an intronic region of the FOXO3 gene and its promoter. The FOXO3 gene is part of the insulin/insulin-like growth factor 1 (IGF-1) signaling pathway (Costales and Kolevzon, 2016). Notably, IGF-I was shown to increase branching and dendritic size in rat primary somatosensory cortex, specifically in pyramidal cells in superficial cortical layers (Niblock et al., 2000). Low IGF-1 levels have also been associated with poor cognitive function during aging (Aleman et al., 1999; Tumati et al., 2016) and a less integrated functional network of connected brain areas (Sorrentino et al., 2017). Thus, individual differences in dendritic elaboration in pyramidal cells are subject to genetic control, go accompanied by functional adaptations in these cells and underlie human variability in intelligence.

How do these findings on cellular and genetic level translate to macroscale findings in brain imaging? One of the most robust finding in brain imaging is that cortical thickness and volume associate with intelligence (Haier et al., 2004; Colom et al., 2006, 2009; Narr et al., 2007; Choi et al., 2008; Karama et al., 2009). Reconstruction of cortical column at nanoscale resolution shows that cortical volume consists largely of dendritic and axonal processes with 7-fold greater number of axons over dendrites (Kasthuri et al., 2015), only a small proportion of this volume is occupied by cell bodies. The dendrites and axons are structures that mediate synaptic plasticity, store information and continue to grow and change during lifetime. Indeed, during normal postnatal development cortical areas follow a similar pattern: dendrites show continuous growth that is accompanied by increased cortical volume and decreased neuronal densities (Huttenlocher, 1990). In addition, frontal cortical areas that are more shaped by age and experience show a slower time course of these changes compared to primary visual areas that have an earlier critical period (Huttenlocher, 1990). In line with this prolonged development, dendritic trees in human temporal lobe continue to grow throughout maturity and into the old age. In 80-year-olds dendritic trees are more extensive than at the age of 50, with most of the difference resulting from increases in the number and average length of terminal segments of the dendritic tree. The link between dendritic size and cognition is emphasized by the fact that in senile dementia, dendritic trees are less extensive, largely because their terminal segments are fewer and shorter (Buell and Coleman, 1979).

Also, within human cortex, a gradient of dendritic complexity exists across cortical areas. Higher order association areas that store and process more complex information contain neurons with larger and more complex dendrites compared to primary sensory areas. At the same time neuronal cell body density is lower in cortical association areas compared to primary sensory areas (Buell and Coleman, 1979; DeFelipe et al., 2002; Elston, 2003).

A recent study by Genç et al. (2018) used multi-shell diffusion tensor imaging to estimate parieto-frontal cortical dendritic density in relation to human cognition. This study found that higher scores in cognitive tests correlated with lower values of neurite density (Genç et al., 2018). As neurite density decreases go together with the increases of dendrite length (Huttenlocher, 1990), the results obtained by Genç et al. (2018) may indicate that parieto-frontal cortical areas in individuals with higher intelligence have less densely packed neurons, and imply that these neurons have larger dendrites. Taking the results of Genç et al. (2018) and Goriounova et al. (2018) together suggests that the neuronal circuitry associated with higher intelligence is organized in a sparse and efficient manner. Larger and more complex pyramidal neurons are more dispersed in cortical space and occupy larger cortical volume.

## Conclusions and Future Perspectives

Brain imaging has provided the basis for research on the neurobiology of intelligence by pointing out important functional and structural gross anatomical regions implicated in intelligence—overall gray matter volume and thickness, white matter integrity and function in temporal, frontal and parietal cortices. However, it is clear that neuroimaging in the present form is unable to provide temporal and spatial resolution sufficient to study the computational building blocks of the brain—neurons and synaptic contacts.

On the other hand, GWAS studies have focused on the other extreme of the spectrum—the genes of intelligence. Large progress was made by increasing sample sizes and combining multiple cohorts. The results show that 98% of the associated genetic variants are not coded into functional protein and probably have a regulatory function at different stages of neural development. However, the small percentage of genes that do produce functional proteins are implicated in various neuronal functions including synaptic function and plasticity, cell interactions and energy metabolism. Importantly, growing database of gene expression profiles has pinpointed the expression of associated genes to principal neurons of cortex and midbrain—pyramidal and medium spiny neurons.

Cellular neuroscience in resected human brain tissue can offer a new perspective. Interesting initial results have already linked pyramidal cell function and structure to human intelligence by revealing positive correlations between dendritic size, action potential speed and IQ. However, many questions still remain unanswered.

What types of neurons are implicated in human intelligence? Recent advances in gene profiling of neurons with single cell resolution indicate that there are around 50 transcriptomic cell types of pyramidal cells in mice and different areas of the brain contain yet new sets of transcriptomic types (Tasic et al., 2018). The information contained in the transcriptomes links the types to their region-specific long-range target specificity. The same can be said about the striatal medium spiny neurons, where the detailed connectivity projection map from the entire cerebral cortex allowed to identify 29 distinct functional domains (Hintiryan et al., 2016). Thus, both pyramidal and medium spiny neurons form very heterogeneous populations, with different cell types having different functions and their specific connectivity patterns with the rest of the brain. How do these mouse cell types correspond to human cell types? How do different cell types support general intelligence and specific cognitive abilities in the human brain? Answers will require large-scale efforts that allow analysis of big numbers, not only of human cohorts, but also of cells and cell types. This may come within reach with the recent large-scale collaborative initiatives that have been started across the globe (Brose, 2016).

## Author Contributions

NG and HM conceptualized the review and wrote the text. NG made the figures.

## Conflict of Interest Statement

The authors declare that the research was conducted in the absence of any commercial or financial relationships that could be construed as a potential conflict of interest.
